# Genomic sequencing of different sequevars of *Ralstonia solanacearum* belonging to the Moko ecotype

**DOI:** 10.1590/1678-4685-GMB-2020-0172

**Published:** 2021-01-08

**Authors:** Ana Karolina Leite Pais, Jessica Rodrigues da Silva, Leandro Victor Silva dos Santos, Greecy Mirian Rodrigues Albuquerque, Antonio Roberto Gomes de Farias, Wilson José Silva, Valdir de Queiroz Balbino, Adriano Márcio Freire Silva, Marco Aurélio Siqueira da Gama, Elineide Barbosa de Souza

**Affiliations:** 1 Universidade Federal Rural de Pernambuco (UFRPE), Departamento de Agronomia, Recife, PE, Brazil.; 2 Instituto Federal de Educação, Ciência e Tecnologia do Piauí (IFPI), Piauí, PI, Brazil.; 3 Universidade Federal de Pernambuco (UFPE), Departamento de Genética, Recife, PE, Brazil.; 4 Universidade Federal de Alagoas (UFAL), Departamento de Agronomia, Maceió, AL, Brazil.; 5 Universidade Federal Rural de Pernambuco (UFRPE), Departamento de Biologia, Recife, PE, Brazil.

**Keywords:** Plant pathogenic bacteria, banana tree, NGS, bacterial wilt, *Musa* spp

## Abstract

Banana vascular wilt or Moko is a disease caused by *Ralstonia solanacearum*. This study aimed to sequence, assemble, annotate, and compare the genomes of *R. solanacearum* Moko ecotypes of different sequevar strains from Brazil. Average nucleotide identity analyses demonstrated a high correlation (> 96%) between the genome sequences of strains CCRMRs277 (sequevar IIA-24), CCRMRs287 (IIB-4), CCRMRs304 (IIA-24), and CCRMRsB7 (IIB-25), which were grouped into phylotypes IIA and IIB. The number of coding sequences present in chromosomes and megaplasmids varied from 3,070 to 3,521 and 1,669 to 1,750, respectively. Pangenome analysis identified 3,378 clusters in the chromosomes, of which 2,604 were shared by all four analyzed genomes and 2,580 were single copies. In megaplasmids, 1,834 clusters were identified, of which 1,005 were shared by all four genomes and 992 were identified as single copies. Strains CCRMRsB7 and CCRMRs287 differed from the others by having unique clusters in both their chromosomes and megaplasmids, and CCRMRsB7 possessed the largest genome among all Moko ecotype strains sequenced to date. Therefore, the genomic information obtained in this study provides a theoretical basis for the identification, characterization, and phylogenetic analysis of *R. solanacearum* Moko ecotypes.

*Ralstonia solanacearum* is a soil-inhabiting plant pathogenic bacterium that is known to infect several economically important crops (Wicker *et al*., 2007), including banana (*Musa* spp.). Upon infecting banana trees, *R. solanacearum* causes vascular wilt, also known as Moko disease. *R. solanacearum* species complex were subdivided into a hierarchical classification system (including phylotypes) based on sequence analysis of the 16S-23S internal transcribed spacer (ITS) region, the endoglucanase (*egl*) gene, and the *mutS* (DNA repair) genes (Prior and Fegan, 2005). Each phylotype was associated with to a geographic origin: phylotype I (Asia), phylotype II (the Americas), phylotype III (Africa), and phylotype IV (Indonesia; Genin and Denny, 2012). Moreover, strains were grouped into sequevars that exhibited variations of ≤ 1% within *egl* gene sequences (Fegan and Prior, 2005).

Nevertheless, since 2014, *R. solanacearum* species complex have been reclassified into three different species according to their phylotypes: *R. pseudosolanacearum* (phylotypes I and III), *R. solanacearum* (phylotype II - IIA and IIB), and *R*. *syzygi* (phylotype IV and closely related strains) (Safni *et al.,* 2014). Among these, the strains that cause Moko disease belong to sequevars IIA-6, IIA-24, IIA-41, IIA-53, IIB-3, IIB-4, and IIB-25 (Cellier and Prior, 2010; Albuquerque *et al.,* 2014; Santiago *et al.,* 2017). 

There are currently 203 publicly-available *R. solanacearum* genome sequences deposited in the National Center for Biotechnology Information (NCBI) database, of which only 17 belong to sequevar isolates IIA-6, IIA-24, IIA-53, IIB-3, and IIB-4 of the Moko ecotype. In Brazil, *R. solanacearum* strains belonging to the Moko ecotype are considered quarantine pests restricted to the northern (Amazonas, Amapá, Pará, Rondônia, and Roraima) and northeastern (Alagoas and Sergipe) states (MAPA, 2018). Although Brazil is likely the biodiversity center of *R. solanacearum* (Santiago *et al*., 2017), there are only three genome sequences of Brazilian strains (IBSBF1900 - IIA-24, IBSBF2570 - IIA-53, and SFC - IIA-53) deposited in the NCBI database. Therefore, our study sought to sequence, assemble, annotate, and compare the genomes of Brazilian *R. solanacearum* strains belonging to sequevars IIA-24, IIB-4, and IIB-25 of the Moko ecotype.

In this study, CCRMRs277 (IIA-24), CCRMRs287 (IIB-4), CCRMRs304 (IIA-24), and CCRMRsB7 (syn. B7; IIB-25) were isolated from Brazilian Amazon banana trees exhibiting Moko disease symptoms. Bacterial strains were cultivated on 2,3,5-triphenyl tetrazolium chloride medium for 48 h at 28 °C and white-colored colonies with pink centers were selected for DNA extraction, which was performed using the PureLink^®^ Genomic DNA Kit (Thermo Fisher Scientific, Waltham, MA, USA) following the manufacturer’s instructions.

For genome sequencing, pair-end DNA library preparation was performed using the Illumina Nextera DNA Flex Prep Kit (Illumina, San Diego, CA, USA) following the manufacturer's recommendations, and sequencing was performed on an Illumina MiSeq-2500 sequencer (100 cycles). Read quality was first assessed with the FastQC software (Andrews, 2010), and sequence trimming was performed using Sickle v.1.33 (Joshi and Fass, 2011). All reads that met the quality control requirements were assembled *de novo* using Unicycler version 3 (Wick *et al.,* 2017) and were then evaluated with Quast v.5.0.2 (Gurevich *et al.,* 2013) to estimate genome size, contig number*,* N50, and GC content (%). Additionally, single-copy orthologs were identified and gene content conservation was analyzed with the BUSCO software (Seppey *et al*., 2019).

The average nucleotide identity (ANI) between assembled genome sequences was obtained via global alignment with the MUMmer algorithm (Kurtz *et al*., 2004) using the Pyani 0.2.7 Python3 module (Pritchard *et al*., 2016). The ABACAS v.1.3.1 software (Assefa et al., 2009) was implemented with the PROmer and NUCmer algorithms (Kurtz *et al*., 2004) to perform chromosome and megaplasmid alignments using the Po82 and UW163 strains (*R. solanacearum* Moko ecotype, sequevar IIB-4) as a reference, as these are the only strains whose whole genomes are deposited in the NCBI database. The QUAST software v.5.0.2 (Gurevich *et al*., 2013) was used to evaluate contigs and select alignment scaffolds with the lowest number of Ns and the largest number of predicted genes. Synteny and visualization of the four *R. solanacearum* genome alignments and the two reference genomes were performed using the Mauve software (Darling *et al*., 2004) and CGView Server (Grant and Stothard, 2008), respectively.

Genome annotation was performed using the RAST online platform (Brettin *et al.,* 2015), which also groups genes into subsystems based on the reconstruction of metabolic systems. The pangenome was built based on data obtained from RAST annotation using the Orthovenn online platform (Wang *et al.,* 2015) to identify clusters of specific genes/orthologs for each strain.

The sequences of the four *R. solanacearum* genomes were assembled into different sizes, of which the CCRMRs287 genome was the smallest (5,464,210 bp) and the CCRMRsB7 genome was the largest (5,847,640 bp) (Table 1). Compared to other Moko ecotype genome sequences available in the NCBI database, strain CCRMRsB7 was found to possess the largest genome sequence identified to date. The coverage between the four genome sequences varied from 129.8x (CCRMRs304) to 163x (CCRMRsB7). Additionally, different strains exhibited considerable variations in assembled contig number and N50 values; however, a gene conservation rate of > 97% was observed in all examined strains, indicating that the assemblies were reliable. Additional genome assembly details are summarized in Table 1.

**Table 1 -:** Characteristics of the genome sequences of four different *R. solanacearum* strains of the Moko ecotype from Brazil.

Features	CCRMRs277^a^		CCRMRs287^b^		CCRMRs304^c^		CCRMRsB7^d^	
Coverage	133		148		129.8		163	
Size before alignment (bp)	5.659.851		5.464.210		5.654.054		5.847.640	
GC content (%)	66.54		66.59		66.55		66.46	
Number of contigs	360		92		368		88	
N50	43.891		150.675		45.590		216.010	
BUSCO (%)	97.97		98.65		97.97		97.97	
Size after alignment (bp)	5.636.326		5.444.697		5.645.239		5.854.658	
	Chrom^e^	Plasmid^f^	Chrom	Plasmid	Chrom	Plasmid	Chrom	Plasmid
References strains	PO82^g^	UW163^h^	UW163^g^	UW163^h^	PO82^g^	UW163^h^	PO82^h^	UW163^h^
Size after alignment (bp)	3.549.795	2.086.531	3.512.030	1.932.667	3.549.663	2.095.576	3.716.474	2.138.184
Coding sequences	3.099	1.718	3.315	1.669	3.070	1.720	3.521	1.750
Subsytems number	299	81	318	83	291	88	321	93
RNAs	46	1	52	1	45	1	51	4

^a^ ^-^ CCRMRs277 = sequevar IIA-24; ^b-^ CCRMRs287 = sequevar IIB-4; ^c-^ CCRMRs304 = sequevar IIA-24; ^d-^ CCRMRsB7 = sequevar IIB-25.

^e^ - Chromosome;

^f^ - Megaplasmid;

^g^ - NUCmer alignment algorithm;

^h^ -PROmer alignment algorithm.

ANIm analysis demonstrated a >96% sequence similarity between the genome sequences, confirming that they belonged to the same species based on an ANIm cut-off value of 95-96% for species delineation (Richter and Rossello-Mora, 2009). Even though the genomes of all strains exhibited high sequence similarity, it was possible to differentiate the strains corresponding to phylotypes IIA (99.9%) and IIB (98.7%). Moreover, even though strain CCRMRsB7 (sequevar IIB-25) belongs to phylotype IIB, it formed a subdivision within this group (Figure 1). Nonetheless, this strain displayed ANIm values of 98% and 96.4% when compared with the other members of phylotypes IIB and IIA, respectively.

**Figure 1 -:**
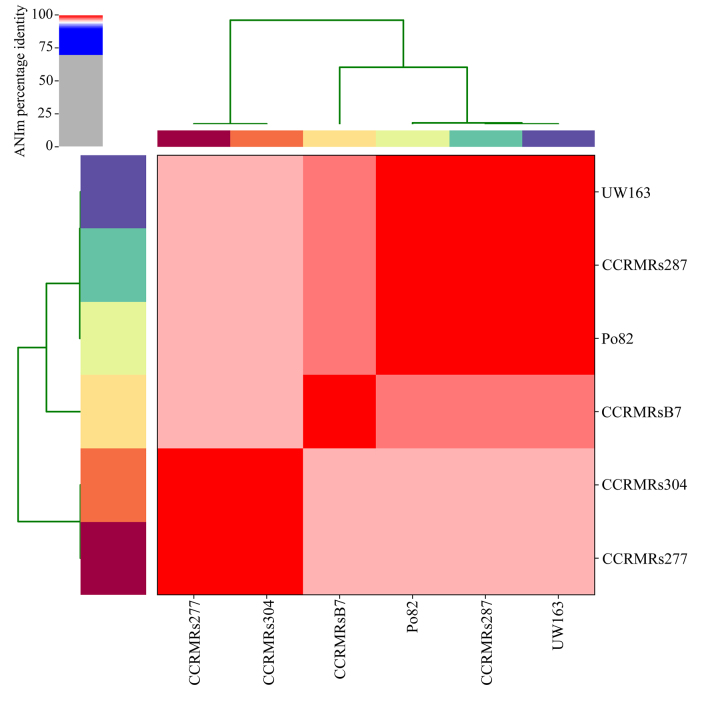
Average Nucleotide Identity (ANIm) of four *R. solanacearum* strains belonging to the Moko ecotype (CCRMRs277, sequevar IIA-24; CCRMRs287, sequevar IIB-4; CCRMRs304, sequevar IIA-24; and CCRMRsB7, sequevar IIB-25) from Brazil, as well as the employed reference strains (UW163 and Po82, sequevar IIB-4).

The *R. solanacearum* complex possesses chromosomes and megaplasmids, and our study determined the lowest number of Ns and the largest number of predicted genes in the replicons. The chromosome scaffolds of CCRMRs277, CCRMRs304, and CCRMRsB7 were formed from alignments with Po82, and the chromosome scaffold of CCRMRs287 and all megaplasmid scaffolds had UW163 as a reference (Table 1).

The synteny of the genomic sequences is illustrated in Figure 2A and 2B, for Po82 and UW163, respectively. In both cases, the linear arrangement of the genomes exhibited a high level of collinearity for the two replicons. However, we also observed translocations and inversions of the locally collinear blocks (LCBs). When Po82 was used as a reference (Figure 2A), inversions were detected in the blue- and violet-colored LCBs and the violet-colored LCB of the CCRMRs287 and CCRMRsB7 strains, respectively. Similarly, when UW163 was used as a reference (Figure 2B), we noted that compared with the other three strains, there were substantial rearrangements in the pink- and green-colored LCBs of the CCRMRs287 strain. We assume that these features are attributable to the evolutionary relatedness of the CCRMRs287 and UW163 (sequevar IIB-4) strains, as well as the proximity of their geographical origins (the cities of Benjamin Constant-BR, and Nauta-PE, respectively, on the border between Brazil and Peru). Although this process has yet to be clarified, it is known to be a common event and can lead to adaptive phenotypic effects, as in the case of transposable elements that carry genes related to antibiotic resistance (Ceccatto, 2015). The circular visualization of the alignment of the genomic sequences produced by CGView showed that most of the genomic regions were highly conserved between the genomes (Figure 2C and 2D).

**Figure 2 -:**
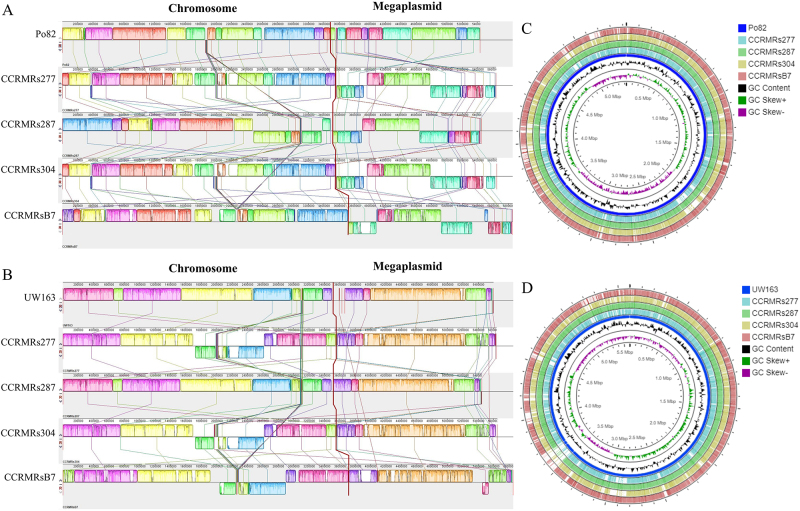
Synteny of four *R. solanacearum* strains belonging to the Moko ecotype (CCRMRs277, sequevar IIA-24; CCRMRs287, sequevar IIB-4; CCRMRs304, sequevar IIA-24; and CCRMRsB7, sequevar IIB-25) from Brazil and reference strains Po82 (A) and UW163 (B). Visualization of the chromosome and megaplasmid alignments of the *R. solanacearum* strains and reference strains Po82 (C) and UW163 (D).

Table 1 details the number of coding sequences (CDS), RNAs, and subsystems of the genome assemblies of strains CCRMRs277, CCRMRs287, CCRMRs304, and CCRMRsB7, as well as the reference genomes and algorithms used for assembly. The chromosomes from the four genomes exhibited higher CDS, RNAs, and subsystem numbers, and therefore contained more information than megaplasmids. This observation may be related to the size and conservation of the chromosome, which represents the larger region (Genin and Denny, 2012) and is more conserved than the megaplasmid (Guidot *et al.,* 2007).

The four most represented subsystems found in the four *R. solanacearum* chromosomes were (I) amino acids and derivatives; (II) protein metabolism; (III) carbohydrates; and (IV) cofactors, vitamins, prosthetic groups, and pigments, which accounted for 49-51% of the total subsystems (Figure 3A). For the megaplasmids, only 15% of the annotated genes were grouped into subsystems of which the most represented were (I) membrane transport; (II) virulence, disease, and defense; (III) carbohydrates; and (IV) nitrogen metabolism, which ranged from 53% to 64% of the total subsystems among the examined strains (Figure 3B ). 

**Figure 3 -:**
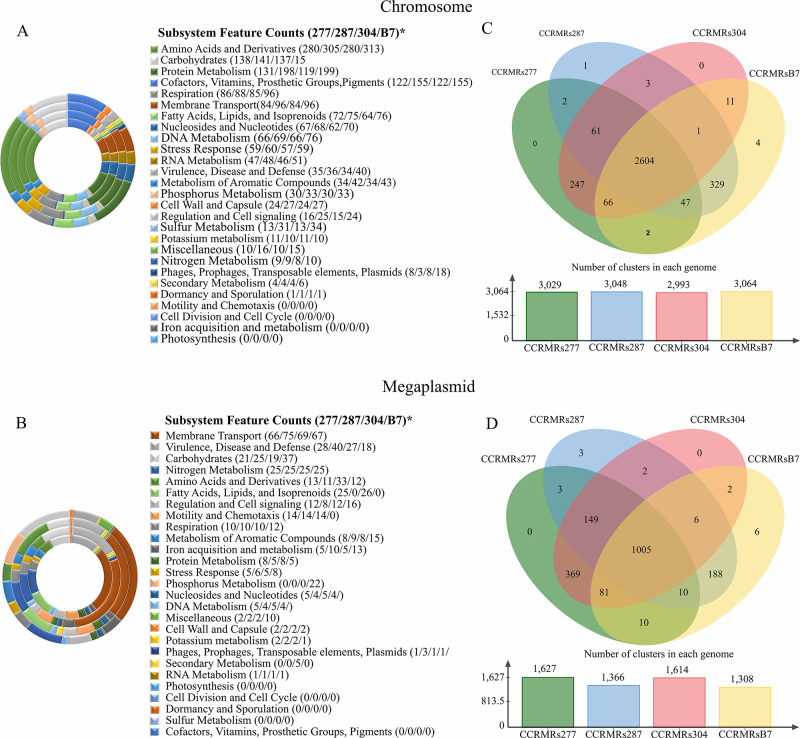
Graphic representation of cluster subsystems of chromosomes (A) and megaplasmids (B) and Venn diagram of the chromosome (C) and megaplasmid (D) clusters of four strains of *R. solanacearum* belonging to the Moko ecotype (*CCRMRs277 = 277, sequevar IIA-24; CCRMRs287 = 287, sequevar IIB-4; CCRMRs304 = 304, sequevar IIA-24; and CCRMRsB7 = B7, sequevar IIB-25) from Brazil, as well as the employed reference strains (UW163 and Po82, sequevar IIB-4).

In the Venn diagram based on the chromosome sequences, 3,378 gene clusters were identified, of which 2,604 were shared by all four genomes analyzed (Figure 3C, top panel), 798 were orthologous, and 2,580 were characterized as single copies. The number of clusters identified in the four sequenced genomes ranged from 2,993 (CCRMRs304) to 3,064 (CCRMRsB7; Figure 3C, bottom panel). Only five clusters were strain-specific, four were specific to strain CCRMRsB7 (sequevar IIB-25), and one to strain CCRMRs287 (sequevar IIB-4); however, none of the clusters were associated with known functions. In the megaplasmid, 1,834 clusters were identified, of which 1,005 were shared by all four genomes analyzed (Figure 3D, top panel), 842 were orthologous, and 992 were identified as single copies. The number of clusters identified in the four genomes ranged between 1,308 (CCRMRsB7) and 1,627 (CCRMRs277; Figure 3D, bottom panel). Nine unique clusters were found; however, only five were associated with known functions, such as peptide transport, antibiotic biosynthesis, cholesterol metabolism (CCRMRsB7), transmembrane transport, and metabolic processes (CCRMRs287).

Among strains of the *R. solanacearum* Moko ecotype sequenced in this study, the genome of CCRMRsB7 is the largest sequenced to date, whereas that of CCRMRs287 is the smallest. However, both genomes are characterized by significant rearrangement of the LCB, which requires further in-depth investigation. Moreover, the replicons of both these strains contain unique clusters. Our results indicate that the genomes of strains CCRMRsB7 (sequevar IIB-25) and CCRMRs287 (sequevar IIB-4) have distinct characteristics compared with other sequenced genomes examined thus far, and accordingly, both strains warrant further analysis in this regard. Moreover, the genomic data elucidated by our study provides a theoretical basis that will facilitate the future identification, characterization, and phylogenetic analysis of the *R. solanacearum* Moko ecotype.

## Genome sequence accession number

The accession numbers are as follows: GCA_014210395.1 for CCRMRs277, GCA_014210375.1 for CCRMRs287, GCA_014210335.1 for CCRMRs304 and GCA_014210345.1 for CCRMRsB7. 

## References

[B1] Albuquerque GMR, Santos LA, Felix KCS, Rollemberg CL, Silva AMF, Souza EB, Cellier G, Prior P, Mariano RLR (2014). Moko disease-causing strains of Ralstonia solanacearum from Brazil extend known diversity in paraphyletic phylotype II. Phytopathology.

[B2] Assefa S, Keane TM, Otto TD, Newbold C, Berriman M (2009). ABACAS: algorithm-based automatic contiguation of assembled sequences. Bioinformatics.

[B3] Brettin T, Davis JJ, Disz T, Edwards RA, Gerdes S, Olsen GJ, Olson R, Overbeek R, Parrello B, Pusch GD (2015). RASTtk: A modular and extensible implementation of the RAST algorithm for building custom annotation pipelines and annotating batches of genomes. Sci Rep.

[B4] Cellier G, Prior P (2010). Deciphering phenotypic diversity of Ralstonia solanacearum strains pathogenic to potato. Phytopathology.

[B5] Ceccatto V M, Ceccatto V M (2015). Variação gênica. Biologia molecular.

[B6] Darling ACE, Mau B, Blattner FR, Perna NT (2004). Mauve: multiple alignment of conserved genomic sequence with rearrangements. Genome Res.

[B7] Fegan M, Prior P, Allen C, Prior P, Hayward AC (2005). How complex is the “Ralstonia solanacearum species complex”. Bacterial wilt disease and the *Ralstonia solanacearum* species complex.

[B8] Genin S, Denny TP (2012). Pathogenomics of the Ralstonia solanacearum species complex. Annu Rev Phytopathol.

[B9] Grant JR, Stothard P (2008). The CGView Server: a comparative genomics tool for circular genomes. Nucleic Acids Res.

[B10] Guidot A, Prior P, Schoenfeld J, Carrere S, Genin S, Boucher C (2007). Genomic structure and phylogeny of the plant pathogen Ralstonia solanacearum inferred from gene distribution analysis. J Bacteriol.

[B11] Gurevich A, Saveliev V, Vyahhi N, Tesler G (2013). QUAST: Quality assessment tool for genome assemblies. Bioinformatics.

[B12] Joshi NA, Fass JN (2011). Sickle: A sliding-window, adaptive, quality-based trimming tool for FastQ files.

[B13] Kurtz S, Phillippy A, Delcher AL, Ml Smoot, Shumway M, Antonescu C, Salzberg SL (2004). Versatile and open software for comparing large genomes. Genome Biol.

[B14] Prior P, Fegan M (2005). Recent developments in the phylogeny and classification of Ralstonia solanacearum. Acta Hortic.

[B15] Pritchard L, Glover RH, Humphris S, Elphinstone JG, Toth IK (2016). Genomics and taxonomy in diagnostics for food security: Soft-rotting enterobacterial plant pathogens. Anal Methods.

[B16] Richter M, Rossello-Mora R (2009). Shifting the genomic gold standard for the prokaryotic species definition. Proc Natl Acad Sci USA.

[B17] Safni I, Cleenwerck I, De Vos P, Fegan M, Sly L, Kappler U (2014). Polyphasic taxonomic revision of the Ralstonia solanacearum species complex: proposal to emend the descriptions of Ralstonia solanacearum and Ralstonia syzygii and reclassify current R. syzygii strains as Ralstonia syzygii subsp. syzygii subsp. nov., R. solanacearum phylotype IV strains as Ralstonia syzygii subsp. indonesiensis subsp. nov., banana blood disease bacterium strains as Ralstonia syzygii subsp. celebesensis subsp. nov. and R. solanacearum phylotype I and III strains as Ralstonia pseudosolanacearum sp. nov. Int J Syst Evol Microbiol.

[B18] Santiago TR, Lopes CA, Caetano-Anollés G, Mizubuti ESG (2017). Phylotype and sequevar variability of Ralstonia solanacearum in Brazil, an ancient centre of diversity of the pathogen. Plant Pathol.

[B19] Seppey M, Manni M, Zdobnov EM, Kollmar M (2019). BUSCO: Assessing genome assembly and annotation completeness. Gene prediction. Methods in Molecular Biology.

[B20] Wang Y, Coleman-derr D, Chen G, Gu YQ (2015). OrthoVenn: a web server for genome wide comparison and annotation of orthologous clusters across multiple species. Nucleic Acids Res.

[B21] Wick RR, Judd LM, Gorrie CL, Holt KE (2017). Unicycler: Resolving bacterial genome assemblies from short and long sequencing reads. PLoS Comput Biol.

[B22] Wicker E, Grassrt L, Coranson-Beaudu R, Mian D, Guilbaud C, Fegan M, Prior P (2007). Ralstonia solanacearum strains from Martinique (French West Indies) exhibiting a new pathogenic potential. J Appl Environ Microbiol.

